# A Fabrication of Multichannel Graphite Electrode Using Low-Cost Stencil-Printing Technique

**DOI:** 10.3390/s22083034

**Published:** 2022-04-15

**Authors:** Supatinee Kongkaew, Suowarot Tubtimtong, Panote Thavarungkul, Proespichaya Kanatharana, Kah Haw Chang, Ahmad Fahmi Lim Abdullah, Warakorn Limbut

**Affiliations:** 1 Center of Excellence for Trace Analysis and Biosensors (TAB-CoE), Prince of Songkla University, Hat Yai, Songkhla 90110, Thailand; supatinee.25@gmail.com (S.K.); panote.t@psu.ac.th (P.T.); proespichaya.k@psu.ac.th (P.K.); 2 Center of Excellence for Innovation in Chemistry, Faculty of Science, Prince of Songkla University, Hat Yai, Songkhla 90110, Thailand; 3 Division of Health and Applied Sciences, Faculty of Science, Prince of Songkla University, Hat Yai, Songkhla 90110, Thailand; malodyaern@hotmail.com; 4 Division of Physical Science, Faculty of Science, Prince of Songkla University, Hat Yai, Songkhla 90110, Thailand; 5 Forensic Science Programme, School of Health Sciences, Universiti Sains Malaysia, Kubang Kerian 16150, Kelantan, Malaysia; changkh@usm.my (K.H.C.); fahmilim@usm.my (A.F.L.A.); 6 Forensic Science Innovation and Service Center, Prince of Songkla University, Hat Yai, Songkhla 90110, Thailand

**Keywords:** multichannel graphite electrodes, stencil-printing technique, nitrite

## Abstract

Multichannel graphite electrodes (MGrEs) have been designed and fabricated in this study. A template was cut from an adhesive plastic sheet using a desktop cutting device. The template was placed on a polypropylene substrate, and carbon graphite ink was applied with a squeegee to the template. The size of the auxiliary electrode (AE) as well as the location of the reference electrode (RE) of MGrEs design were investigated. Scanning electron microscopy was used to determine the thickness of the ink on the four working electrodes (WEs), which was 21.9 ± 1.8 µm. Cyclic voltammetry with a redox probe solution was used to assess the precision of the four WEs. The intra-electrode repeatability and inter-electrode reproducibility of the MGrEs production were satisfied by low RSD (<6%). Therefore, the MGrEs is reliable and capable of detecting four replicates of the target analyte in a single analysis. The electrochemical performance of four WEs was investigated and compared to one WE. The sensitivity of the MGrEs was comparable to the sensitivity of a single WE. The MGrEs’ potential applications were investigated by analyzing the nitrite in milk and tap water samples (recoveries values of 97.6 ± 0.4 to 110 ± 2%).

## 1. Introduction

The development of single channel, dual channel, and multichannel sensors has attracted attention in clinical analysis [1,2,3,4,5], food analysis [6], and environmental analysis [7] applications. The increasing interest is driven by the high accuracy and faster response of multichannel sensing for the electroanalytical method. Several advantages of electroanalytical methods have been admitted, such as simplicity and low cost [8,9]. Multichannel sensors used for electrochemical detection (ECD) have especially advanced properties that enable the determination of several analytes at the same time or simultaneous measurements of a single analyte.

A multichannel sensor has been fabricated in which each working electrode has its own reference and auxiliary electrode [10]. In this case, multiple electrodes of three combined on the same platform can detect multiple analytes with the individual system. In order to miniaturize the size and reduce the complexity of the electrochemical cell, sharing the reference and auxiliary electrodes with multiple working electrodes is one option for fabricating the multi-sensors [11,12]. Various fabrication strategies have been proposed for fabrication of multichannel sensors for ECD. These strategies have included electrospinning [13], inkjet printing [14], screen printing [15], and micro-electro-mechanical-system (MEMS) technologies [16]. Multichannel sensors for ECD typically use disposable electrodes based on carbon materials. The most common advanced carbon materials are conductive carbon paper and conductive carbon ink [17,18]. These materials are chosen for their cost-effectiveness, good electrical conductivity, and availability in a variety of allotropic forms. They produce low background currents and wide potential windows, and their easily renewable surfaces can be used in unmodified and modified forms [19,20].

Graphite is a prevalent form of carbon material used for electrochemical sensors, which is available in a variety of formats such as ink [21,22] and paper [23]. In addition, it has a low price and good conductivity [24]. The bendable and flexible application has received considerable attention for the next-generation of analytical sensors. Polypropylene (PP) is one of the most affordable plastics on the market and is low cost. PP has good properties, including smoothness, easy modification, flexibility, low moisture adsorption, and good chemical and temperature resistance [25,26,27,28,29,30]. The established approach to the fabrication of flexible electrodes involves a screen-printing technique. The procedure is simple and enables mass production of small devices with high reproducibility and low cost [31,32]. The screen-printing technique requires the production of a template, which is normally made of polyester mesh stretched and attached to a screen frame made of wood or metal block. This kind of template deposits a homogeneous layer of conductive ink on the substrate since the ink is filtered through the mesh [33,34]. However, this kind of template needs a screen frame and a special chemical to make positive and negative masks. Another method for creating a simple template is to cut out and discard the desired pattern while leaving the unwanted part of the template attached to the substrate, which is known as the stencil printing technique. Stencil printing is a variant of screen printing in which conductive ink is transferred to the substrate using a squeegee. The ink forms the pattern of the template on the substrate [35,36]. This method is simpler and eliminates the problem of mesh screen clogging, which hinders the reproducibility of electrode production. Despite its advantages, stencil printing has a low resolution, requiring the use of predesigned masks. However, this method is also a good choice for making the screen-printed electrode because it is simple, easy to make, uses cheap materials, and can be used for large-scale production.

The practical performance of unmodified carbon based-electrodes has been presented [37,38,39] including nitrite [40]. A nitrite is a simple inorganic compound that can be reduced to another compound by an applied potential. It is present in industrial and agricultural settings, either by intended or unintended introduction. Unfortunately, nitrite can react to amines or amides in the human body and give rise to carcinogenic compounds. The safe amount of nitrite has been set at different concentrations: 2 ppm in milk products [41], 150 mg kg^−1^ in meat products [42], and 3 ppm in drinking water [43]. Therefore, it is imperative to detect nitrites with a fast and accurate tool. Numerous modified materials have been proposed for nitrite detection by electrochemical sensors. They have included carbon materials [44,45] and metal composites [46,47,48]. Inks containing graphite or carbon not only have good electrocatalytic activity but can also absorb the target analytes via oxygen functional groups [49]. As a result, the introduction of graphite-based ink can work for the construction of nitrite sensors, which are used as an analyte model for this work.

There is an emphasis on research, new designs, and fabrication of electroanalytical sensing platforms. In general, the use of an electrochemical system with the bulk volume cell that consists of one reference electrode, one working electrode, and one auxiliary electrode is widely accepted. This paper proposes the development of a multichannel electrochemical sensor using screen/stencil printing. The four working electrodes (WEs; WE1, WE2, WE3, and WE4) shared one auxiliary electrode (AE) and one reference electrode (RE) in the multichannel device. Four working electrodes in MGrEs have been fabricated due to the standard replication of the analytical technique being admitted to three replications (n = 3). Using the MGrEs platform, we achieved four replications (n = 4) in a single measure with a reduction in reagent consumption. To make the device disposable and flexible, the polypropylene sheet (PP) was used as a substrate for the electrode platform. A template was constructed with a desktop cutting device and graphite-carbon ink was screened through the template to form the multichannel graphite electrodes (MGrEs). The position of the RE and size of the AE were studied using a redox probe solution. The performance of the appropriate design of the MGrEs was tested using a nitrite model analyte. The proposed MGrEs was further tested with real milk and tap water samples to determine the device’s practical application.

## 2. Materials and Methods

### 2.1. Reagents

Potassium chloride (KCl) was obtained from Merck (Darmstadt, Germany). Potassium ferrocyanide (K_4_[Fe(CN)_6_]), potassium ferricyanide (K_3_[Fe(CN)_6_]), and sodium nitrite (NaNO_2_) were obtained from Sigma-Aldrich (St. Louis, MO, USA). Potassium hydrogen phosphate (K_2_HPO_4_) and potassium dihydrogen phosphate (KH_2_PO_4_) were obtained from Ajex Fine Chem Pty Ltd. (Auckland, New Zealand). All chemicals were analytical grade. Deionized water (Barnstead Mega-Pure, resistivity ~18 MΩ cm, Thermo Fischer Scientific^TM^, Marietta, OH, USA) was used to prepare all solutions. Carbon graphite ink (serial No. C2050106P7) and silver/silver chloride paste ink (serial No. C2140310d1) were from Gwent Electronic Materials Ltd. (Gwent, UK). Polypropylene card and vinyl adhesive sheet were obtained from a local stationery store (Hatyai, Songkhla, Thailand).

### 2.2. Instrumentation and Measurements

All electrochemical experiments were carried out using a bipotentiostat/galvanostat (Model μStat 400, DropSens S.L., Asturias, Spain). A desktop cutting machine (Silhouette CAMEO version 2) was used to cut the stencil mask from polypropylene sheet. A digital multimeter (YUGO industrial, Bangkok, Thailand) was used to prior test the resistance of all electrodes. Surface and cross-sectional morphologies were observed by scanning electron microscope (Model Quanta 400, Thermo Fisher Scientific, FEI, USA). The electrochemical system consisted of the multi-electrodes platform (four WEs (WE1, WE2, WE3, and WE4), one pseudo-RE, and one AE), a lab-made electrochemical connector, and a beaker (25 mL) served as an electrochemical container. The electrochemical behavior and effect of layout design were studied by CV in 0.1 M potassium chloride (KCl) solution containing various different concentrations of redox solution (Fe(CN_6_)^3−/4−^) at room temperature. The application on nitrite detection was investigated using differential pulse voltammetry technique in 0.1 M phosphate buffer (PB) solution pH 7.0 containing different concentrations of nitrite (condition carried out; Edep = −0.5 V, tdep = 15 s, Estep = 0.01 V, Epulse = 0.25 V, tpulse = 100 ms, Srate = 0.04 Vs^−1^).

### 2.3. Electrode Construction

**Figure 1:**
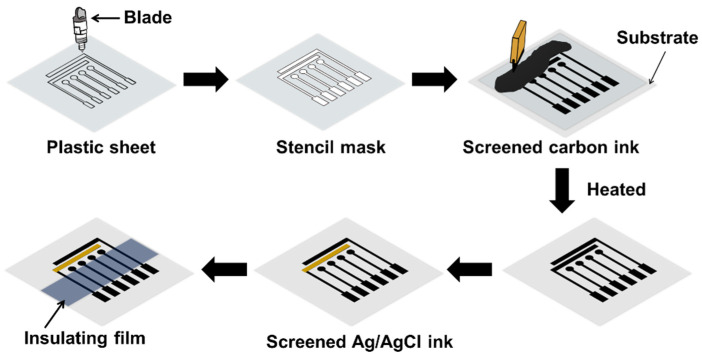
Schematic illustration of the multichannel graphite electrodes fabrication.

The multichannel graphite electrodes were constructed as follows. The layout of the electrode platform was drawn by Silhouette studio software version 4.3. The platform consisted of four WEs, a pseudo-RE, and an AE. The layout design was cut into vinyl adhesive sheet with the cutting device. After cutting the layout pattern, the unwanted parts were peeled off with tweezers, leaving the electrode design on the release liner, forming a stencil template. The stencil electrode layout was attached to the PP sheet with adhesive tape and was filled with carbon graphite ink using a squeegee. The PP sheet was cured at 70 °C for 15 min to evaporate the solvent from the ink and the stencil mask was removed. The same procedure was performed to fabricate the pseudo-RE, but this time PP sheet was used to form the mask. Ag/AgCl ink was spread on the dried carbon ink at the appropriate place and the platform was cured again at 70 °C for 15 min. To complete the fabrication of the electrode, a polypropylene insulating film was covered on top of the multichannel graphite electrodes to define the sensing area (Figure 1). The complete MGrEs was kept in the sealing/desicator box at room temperature.

## 3. Results and Discussion

### 3.1. Studied the Fabrication of MGrEs Platform

**Figure 2:**
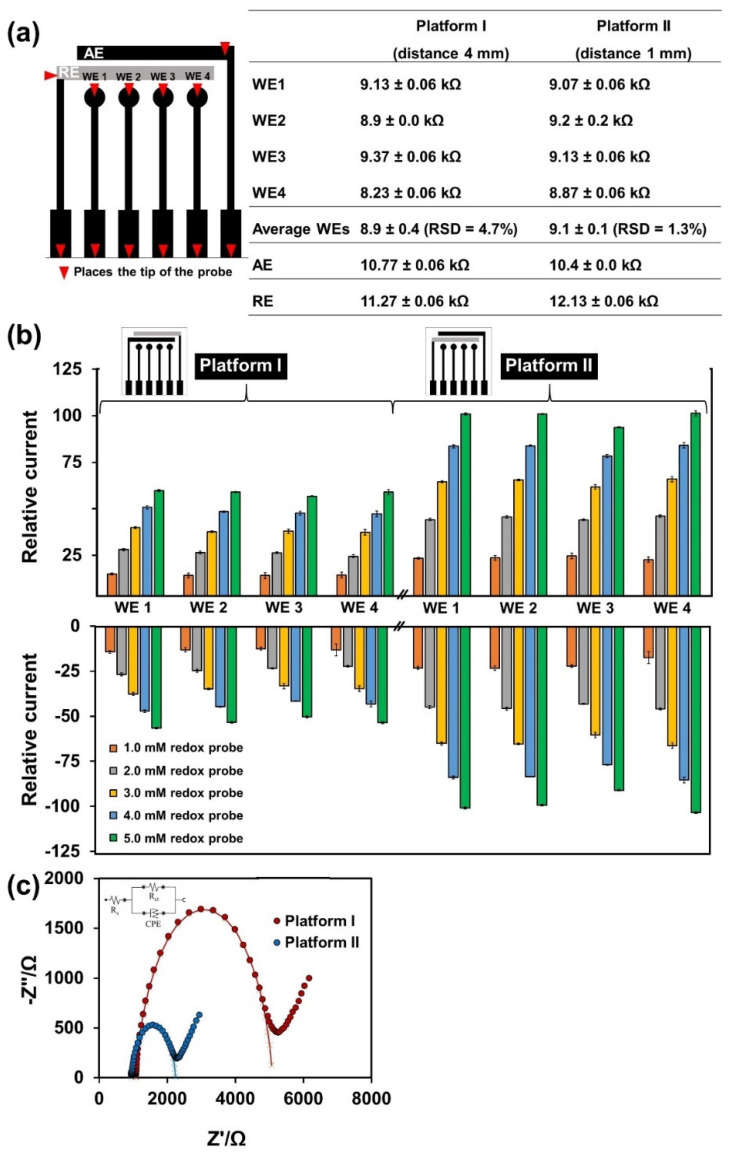
(**a**) The diagram showed the position for resistance measuring using digital multimeter and the table showed the resistance value of two different MGrEs with reference electrodes placed in different locations. (**b**) Histograms of the relative responses of the anodic peak current and cathodic peak current obtained from platform I (distance 4 mm) and platform II (distance 1 mm) in 0.1 M KCl solution containing series concentrations of Fe(CN_6_)^3−/4−^. (**c**) The EIS response of 5.0 mM of Fe(CN_6_)^3−/4−^ obtained from different two platforms.

According to our design of the MGrEs platform as shown in Figure 1, the distance between 4WEs and sharing single RE was studied using two layouts (1 mm and 4 mm), where the RE was located at different places. The layouts were labeled as platform I (distance 4 mm) and platform II (distance 1 mm) (Figure 2b inset). Prior to this, the resistance of the WEs of the two platforms was first checked by digital multimeter (Figure 2a) and the two platforms were then used to measure the anodic and cathodic peak currents in 0.1 M KCl solution containing series concentrations of the Fe(CN_6_)^3−/4−^ by CV. The resistance values of 4WEs in the platform I and platform II were 8.9 ± 0.4 kΩ (RSD = 4.7%), and 9.1 ± 0.1 kΩ (RSD = 1.3%), respectively, which showed no different resistance of two platforms (Figure 2a). The AE and RE resistances of the two platforms (I and II) were also checked using a digital multimeter; the values were 10.77 ± 0.06 kΩ, and 11.27 ± 0.06 kΩ for platform I and 10.4 ± 0.0 kΩ, and 12.13 ± 0.06 kΩ for platform II, respectively. These results confirm that the connection of each electrode of multichannel graphite electrodes of both platforms, which were fabricated using the stencil printing method, was properly connected to each electrode. The anodic peak current and cathodic peak current (Figure 2b) obtained from these two platforms were compared to assess the influence of the distance between WE and RE. The relative responses of the anodic peak current obtained from platform II (distance 1 mm) were higher than the value obtained from platform I (distance 4 mm) about 1.5, 1.6, 1.7, 1.7, 1.7 folds for Fe(CN_6_)^3−/4−^ concentration of 1.0, 2.0, 3.0, 4.0, 5.0 mM, respectively. The relative responses of the cathodic peak current obtained from platform II (distance 1 mm) were higher than the value obtained from platform I (distance 4 mm) about 1.6, 1.8, 1.8, 1.8, 1.8 folds for Fe(CN_6_)^3−/4−^ concentration of 1.0, 2.0, 3.0, 4.0, 5.0 mM, respectively. The results showed that the platform II (distance 1 mm) provided a higher current signal compared to platform I (distance 4 mm) (Figure 2b). The difference in current response can be explained in terms of internal resistance drop (iR drop) or ohmic potential drop. In an electrochemical system, a varying potential is applied to the WE and compared with the constant potential at the RE. Furthermore, the EIS experiment was performed on two platforms in 0.1 M KCl containing 5.0 mM Fe(CN_6_)^3−/4−^. The diameter of the semicircle in the fitted curves was used to evaluate the charge transfer resistance (Rct) of WE on the two platforms. The result showed that, the Rct obtained from platform II (Rct = 1.4 ± 0.2 kΩ) was lower than platform I (Rct = 2.8 ± 0.1 kΩ), which was shown in the example in Figure 2c. Since the configuration of platform II provides a shorter distance (1.0 mm) between the RE and WE than that of platform I (distance between WE and RE = 4.0 mm). The distance between the RE and the WE might increase the iR drop or cause a loss of potential, which translates into increased peak-to-peak separation or reduced current signal [50]. As a result, the platform II (distance between WE and RE = 1 mm) provided the more suitable layout for our design for multichannel sensing.

**Figure 3:**
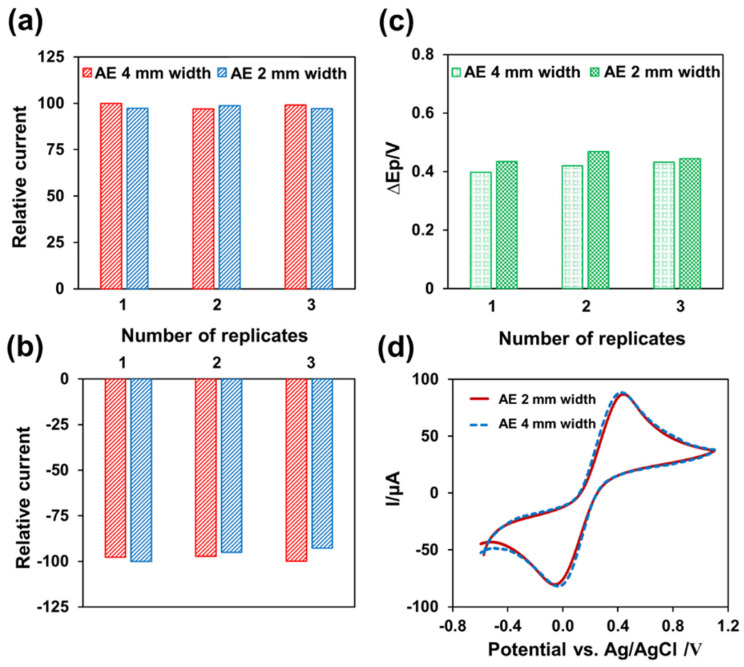
Histograms of the relative responses of the anodic peak current (**a**) and cathodic peak current (**b**) obtained from AE of different sizes (2 mm and 4 mm width) on the MGrEs platform II (distance between WE and RE = 1 mm). Peak-to-peak separation obtained from AE of different sizes (2 mm and 4 mm width) on the MGrEs platform II using 5.0 mM Fe(CN_6_)^3−/4−^ (**c**). An example of cyclic voltammograms obtained from various sizes of AE (solid line: 2 mm width and dotted line: 4 mm width) (**d**).

Based on the multichannel graphite electrodes consisted of sharing RE and AE, the size of the AE constructed by graphite ink might affect the electrochemical signal. Therefore, two MGrEs layouts were used to studied by fixed the distance between WE and RE: one with an AE 2 mm width and another with an AE 4 mm width. The electrochemical signal was measured with both layouts (Figure 3a). The relative current responses of the Fe(CN_6_)^3−/4−^ solution were calculated by normalizing the highest current obtained from each reaction at the MGrEs’ surface. The relative responses of the anodic peak current were 92–100% for both AE sizes (Figure 3a), while the relative responses of the cathodic peak current were 97–100% (Figure 3b). Figure 3d shows an example of cyclic voltammograms obtained from different sizes of AE (solid line: 2 mm width and dot line: 4 mm width). As a result, there was no significant difference in anodic and cathodic peak current with ∆Ep of 0.32–0.33 V at 5.0 mM of Fe(CN_6_)^3−/4−^ concentration (Figure 3c). According to our findings, increasing the area of the AE of MGrEs has no significant effect on the electrochemical signal. As a result, the 2 mm AE was chosen for the MGrEs design.

### 3.2. Characterization of MGrEs

**Figure 4:**
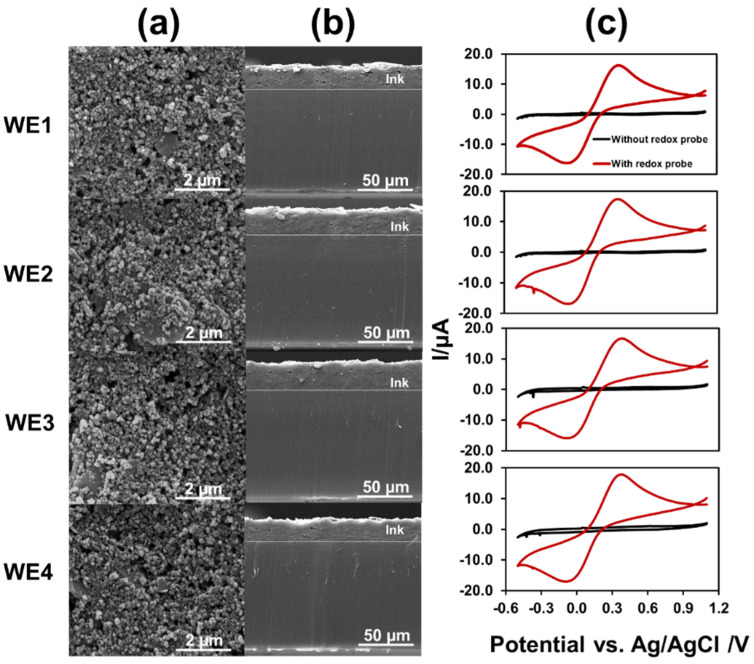
Surface morphologies (**a**) and cross-sections (**b**) of 4WEs were studied using scanning electron microscopy. The cyclic voltammograms of the MGrEs in 0.1 M KCl without (black line) and with 1.0 mM Fe(CN_6_)^3−/4−^ (red line) (**c**).

The morphology of the MGrEs was studied by SEM and the electrochemical behavior was characterized by CV. The carbon graphite ink was uniformly dispersed on the substrate (Figure 4a) at an average thickness of 21.9 ± 1.8 µm (20.1 ± 0.5, 20.6 ± 0.4, 23.6 ± 0.1, and 23.2 ± 0.4 μm for WE1, WE2, WE3, and WE4, respectively). The thickness measurements were obtained from cross-sectional SEM images (Figure 4b). The finding showed that the four WEs were composed of similar amounts of carbon material. As a result, the preparation of the multielectrode platform was efficient. The multielectrode platform was electrochemically characterized by CV in 0.1 M KCl containing 1.0 mM Fe(CN_6_)^3−/4−^ (Figure 4c). The electroactive surface area of the WE was calculated using the Randles–Sevcik equation, Ipa = 2.69 × 10^5^ n^3/2^ A C_0_ D^1/2^ *v*^1/2^, where Ipa is anodic peak current, n is the number of electrons transferred, (A is the surface area of the electrode, cm^2^), (D is the diffusion coefficient, C_0_ is the concentration of Fe(CN_6_)^3−/4−^, and *v* is the scan rate, V s^−1^). The active surface areas of the four WEs were 9.7 ± 0.2, 9.7 ± 0.6, 9.7 ± 0.4, and 9.7 ± 0.1 mm^2^ (RSD = 0.4%) calculated from the anodic peak current. The results demonstrated that the lab-made MGrEs had excellent precision.

The electrochemical kinetics at the surface of the MGrEs were studied by CV, cycling at different scan rates in the Fe(CN_6_)^3−/4−^ solution. The redox peak current increased with increasing scan rate from 25 to 450 mV s^−1^ and exhibited a more linear correlation to the square root of the scan rate than to the scan rate. The linear regression equations for WE1, WE2, WE3, and WE4 were Ipa = (131 ± 3)*v*^1/2^ + (16 ± 1) (R^2^ = 0.9942), Ipa = (136 ± 3)x + (15 ± 1) (R^2^ = 0.9940), Ipa = (130 ± 5)x + (16 ± 2) (R^2^ = 0.9833), and Ipa = (145 ± 4)x + (14 ± 2) (R^2^ = 0.9919), respectively. The result indicated that the electrochemical mechanism of the Fe(CN_6_)^3−/4−^ at the MGrEs was controlled by the diffusion process in a scan rate range from 25 to 450 mV s^−^^1^. The relationship between the logarithm of current and the logarithm of scan rate was also investigated. The slope of the plot was close to the theoretical value (0.5), suggesting that the kinetics reaction of the Fe(CN_6_)^3−/4−^ at the MGrEs was mainly a diffusion-controlled process.

### 3.3. Electrochemical Performance of MGrEs

**Figure 5:**
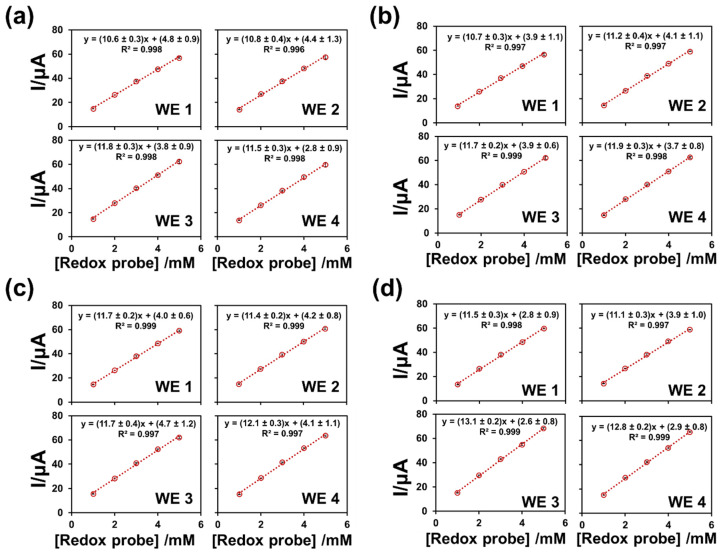
The calibration plot of different concentration of Fe(CN_6_)^3−/4−^ at different four platforms were obtained from MGrE1 (**a**), MGrE2 (**b**), MGrE3 (**c**), MGrE4 (**d**) carried in 0.1 M KCl.

Since the electrochemical behavior of the multichannel platform was satisfactory, the analytical performances of the platform were then evaluated. Precision was a crucial parameter because the MGrEs was fabricated in the lab without advanced equipment. The precision of the device was evaluated in terms of intra-electrode repeatability and inter-electrode reproducibility. The intra-electrode repeatability of a single MGrEs fabrication was evaluated by measuring the anodic peak current response of three concentrations of Fe(CN_6_)^3−/4−^ (1.0, 2.0, and 3.0 mM, n = 3) throughout all six different batches (total 54 measurements). The anodic peak current response was measured, and the relative standard deviation (RSD) of the response was used to determine the precision of the electrode platform. The RSD of anodic peak current response was in the range of 1.0–5.9%, which is acceptable according to the AOAC guideline [51]. The CV response was similar for all four WEs on the same MGrEs, with a low RSD (6%). As a result, one MGrEs fabrication could detect at least six batches with good repeatability. The inter-electrode reproducibility of the method was investigated using four MGrEs fabricated on the same day using the same procedure. The MGrEs were used to measure a series concentration of the Fe(CN_6_)^3−/4−^ (1.0–5.0 mM) in 0.1 M KCl solution. The calibration plots of four different MGrEs were used to calculate the sensitivity of response (Figure 5). The linear regression of the sixteen WEs demonstrated similar results, with a good coefficient of determination. For the purpose of demonstrating the precision of the results, all data were subjected to an analysis of variance (ANOVA) and the *F* value was compared. The *F* value obtained from ANOVA indicates that the individual observations in each group are different from each other when compared to the variation of the individual observations [52]. The *F* value from the sixteen WEs (1.6) was less than the *F_critical_* value (3.5) at α error level 0.05, which revealed no significant difference between the four MGrEs fabricated (sixteen electrodes). As a result, the proposed stencil/screen printing technique demonstrated excellent reliability and produced multielectrode devices with excellent electrode preparation reproducibility.

### 3.4. The Application of the MGrEs

**Figure 6:**
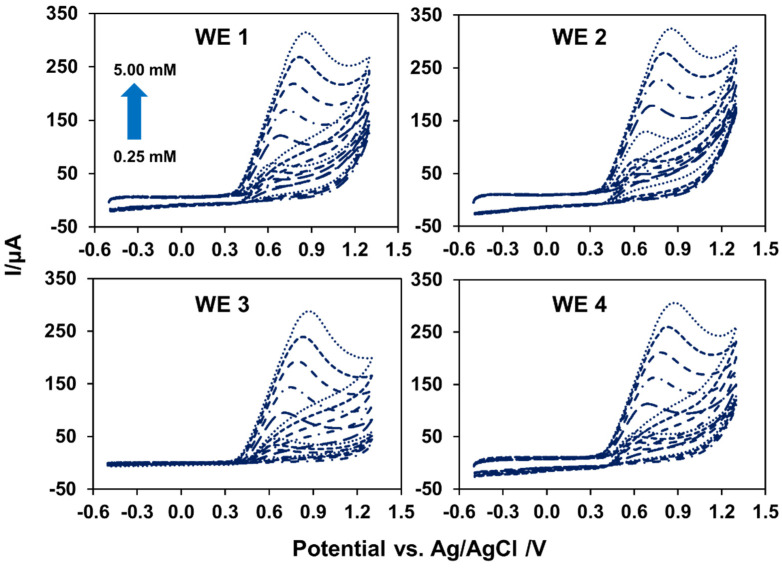
The CVs of nitrite obtained from four WEs on the proposed MGrEs at the same time in the concentration range of 0.25 to 5.00 mM.

To highlight the benefits of the proposed MGrEs, the performance of the proposed MGrEs for use in the sensor was investigated using a nitrite model analyte. Two studies were carried out: (i) the performance of MGrEs in nitrite detection, and (ii) the application of the analysis to nitrite. In the cast performance of MGrEs toward nitrite detection, four WEs on the same platform (MGrEs: WE1, WE2, WE3, and WE4 shared one RE and one AE in the multichannel device) were compared to each single WE vs. RE and AE (which is normally used in electrochemical analysis). Two experiments were designed to detect nitrite in 0.1 M PB at pH 7.0. In the first experiment, nitrite concentrations ranging from 0.25 to 5.00 mM were measured at all four WEs on the MGrEs at the same time (Figure 6). In the second experiment, a single WE was used to measure the same concentration’s range of nitrite for four batches. Both experiments demonstrated an increase in anodic peak current as nitrite concentration increased, with the same linear range of 0.25 to 2.00 mM. The sensitivity of four WEs on the MGrEs to simultaneous nitrite detection was no different from the sensitivity of a single WE for four batches (Table 1), as determined by *t*-test (*t* stat *t* critical). It should be noted that the RSD value obtained from different single WE from different four batches was 4.1%, which is higher than the RSD value obtained from four WEs on the MGrEs detected at the same time in the single batch (RSD = 2.9%). For analytical detection, quantitative measurements should have at least three replicates to express the accuracy of the result [53]. The electrochemical kinetics of nitrite at the MGrEs were then investigated using a scanning potential of 0.025 to 0.450 V s^−1^. The linearity of the peak current vs. scan rate plot, or the linearity of the peak current vs. the square root of the scan rate plot, determined whether the process was controlled by adsorption or diffusion [54]. The anodic peak current increased more linearly with the increasing scan rate. The linearity of the plot indicated that the electrochemical reaction of nitrite at the MGrEs surface was an adsorption-controlled process, which was in agreement with previous reports [55,56].

**Figure 7:**
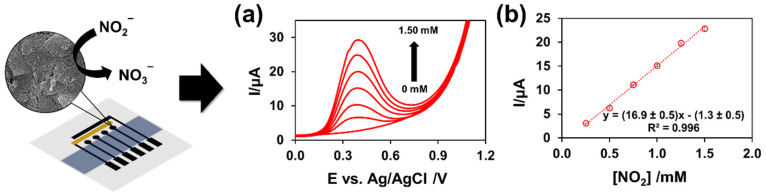
An example of the DPVs of standard nitrite concentration from 0 to 1.50 mM carried in 0.1 M PB at pH 7 (**a**) and the standard curves were obtained from the proposed electrode fabrication (**b**).

For the purpose of applying the analysis to nitrite, differential pulse voltammetry (DPV) was used in the samples (i.e., tap water and milk product samples). First, a nitrite standard solution was evaluated in two steps: an accumulation step with a −0.5 V accumulation potential and a 15 s accumulation time, and a stripping step with the signal recorded during the stripping step. The differential pulse voltammograms indicated that the anodic peak current increased proportionally to nitrite concentration, with the peak potential occurring at 0.38 V. (Figure 7). The effect of interferences was studied prior to the analysis of the real sample using 0.5 mM of nitrite and mixing with other compounds. The current change was measured after 10 times adding K^+^, Na^+^, Ca^2+^, NO_3_^−^, SO_4_^2−^, and Cl^−^ compared to the initial nitrite response. When measuring 0.5 mM of nitrite mixed with other compounds, the results showed that the current changed by less than 5%. These findings suggest that the MGrEs could be used to determine nitrite in a real sample. To validate the multichannel sensor, the MGrEs was used to determine nitrite in tap water and milk product using the standard addition method. The standard curve was previously constructed by detecting a known concentration of standard nitrite. For real samples, the buffer solution (0.1 M PB at pH 7.0) was used to dilute the samples of tap water and milk products. After that, the samples were spiked with the standard nitrite solution, and the accuracy was evaluated. The recovery percentage was calculated using the equation %recovery = ((C_F_ − C_U_)/C_A_) × 100, where C_F_ is the concentration of fortified or spiked samples, C_U_ is the concentration of unfortified samples or blank, and C_A_ is the concentration of standard analyte that is added to the samples. Table 2 provides a summary of the findings. Recoveries ranged from 97.6 ± 0.4 to 110 ± 2% with an RSD of 3.8%, indicating that the MGrEs have the potential to be used to determine nitrite in samples. The MGrEs would be an alternative platform for nitrite. In the future, it may be possible to develop a simultaneous electrochemical sensor designed to detect multiple analytes.

## 4. Conclusions

A stencil template and printing technique were used to design and fabricate multichannel graphite electrodes (MGrEs). Four working electrodes were created using graphite carbon ink, with one auxiliary electrode and one reference electrode shared. Electrochemical behavior of the MGrEs fabrication showed that when the distance between the reference electrode to the working electrode was shortest (platform II; distance between WE and RE = 1 cm), the peak currents and peak-to-peak separation responses improved. In either case, the AE size had no effect on the electrochemical responses. Based on the appropriate design, the MGrEs demonstrated good precision between electrodes on the same platform and between platforms, with an RSD < 6%. The use of graphite carbon ink screened on a plastic sheet for multichannel electrodes not only enables a flexible device, but also improves the precision (four replicates) of the target analysis in a single analysis. The MGrEs displayed good accuracy (recoveries 97.6 ± 0.4 to 110 ± 2%) for detecting nitrite in milk and tap water samples. The proposed MGrEs platform could be used to create electrochemical sensors for target analytes of interest in environmental, food, and forensic analysis.

## Figures and Tables

**Table 1:** The linear regression of anodic peak current of nitrite (0.25–2.00 mM) obtained from four WE of an MGrEs and a single WE tested by CV.

Electrodes	Linear Regression (Ip_a_)	R^2^
Single MGrEs (consisted of 4WEs)	y = (29.7 ± 0.6)x + (1.1 ± 0.7)	0.9986
	y = (30.3 ± 0.5)x + (0.7 ± 0.6)	0.9991
	y = (31.6 ± 0.6)x + (1.0 ± 0.6)	0.9990
	y = (31.9 ± 0.3)x + (0.1 ± 0.4)	0.9997
Single WE	y = (30.8 ± 0.7)x − (0.4 ± 0.8)	0.9985
	y = (33.4 ± 0.7)x − (1.2 ± 0.8)	0.9987
	y = (32.6 ± 0.6)x − (1.6 ± 0.7)	0.9988
	y = (34.5 ± 0.6)x − (2.4 ± 0.7)	0.9990

**Table 2:** Determination of nitrite in tap water and milk product samples and the recoveries.

Samples	Added (mM)	Found (mM)				Recovery (%)				RSD (%)
		WE1	WE2	WE3	WE4	WE1	WE2	WE3	WE4	
Tap water	-	-	-	-	-	-	-	-	-	-
	0.50	0.52 ± 0.01	0.488 ± 0.002	0.517 ± 0.007	0.512 ± 0.006	105 ± 3	97.6 ± 0.4	103 ± 1	102 ± 1	3.1
	0.75	0.794 ± 0.03	0.759 ± 0.005	0.82 ± 0.02	0.79 ± 0.03	106 ± 3	101.2 ± 0.7	109.4 ± 0.5	106 ± 4	3.8
Milk product	-	-	-	-	-	-	-	-	-	-
	0.75	0.79 ± 0.02	0.81 ± 0.004	0.79 ± 0.03	0.83 ± 0.02	106 ± 2	108 ± 2	106 ± 4	110 ± 2	3.0
	1.00	1.01 ± 0.02	1.04 ± 0.01	1.04 ± 0.01	1.07 ± 0.02	101 ± 2	103.6 ± 0.8	104 ± 1	107 ± 3	2.8

## Data Availability

The data presented in this study are available on request from the corresponding author.
